# Nursing home residents with suspected urinary tract infections: a diagnostic accuracy study

**DOI:** 10.1186/s12877-022-02866-2

**Published:** 2022-03-07

**Authors:** Katrien Latour, Jan De Lepeleire, Boudewijn Catry, Frank Buntinx

**Affiliations:** 1 Department of Epidemiology and Public Health, Sciensano, Brussels, Belgium; 2 Department of Public Health and Primary Care, KU Leuven, Leuven, Belgium; 3 Faculty of Medicine, Université Libre de Bruxelles, Brussels, Belgium; 4 Department of General Practice, Maastricht University, Maastricht, The Netherlands

**Keywords:** Urinary tract infections, Point-of-care testing, Long-term care, Aged

## Abstract

**Background:**

Urinary tract infections (UTIs) are one of the most common infections in nursing homes (NHs). A high error rate of a UTI diagnosis based solely on clinical criteria is to be expected in older persons as they often present infections in an atypical way. A study was set up to assess the diagnostic value of signs/symptoms and urine dipstick testing in identifying UTIs in NH residents and to explore whether C-reactive protein (CRP) measured by point-of-care testing (POCT) can help in the diagnosis.

**Methods:**

During a three month prospective multicentre study, urine sampling for culture, POCT CRP and urinary dipstick testing were performed in each NH resident with a suspected UTI. UTIs were defined according to Stone et al., i.e. criteria based upon the presence of a set of signs/symptoms and a positive urine culture.

**Results:**

Eleven NHs and 1 263 residents participated. Sixteen out of 137 recorded UTI suspicions were confirmed. Acute dysuria (positive likelihood ratio (LR +): 7.56, 95% confidence interval (CI): 3.94–14.5) and acute suprapubic pain (LR + : 11.4, 95% CI: 3.58–35.9) were found to be significant predictors. The combined nitrite and leucocyte esterase urine dipstick test (one or both positive) had a 96.0% negative predictive value (95% CI: 80.5–99.3%). The sensitivity of a positive CRP test (≥ 5 mg/L) was 60.0% (95% CI: 32.3–83.7%). Antimicrobials were prescribed in 60.2% of suspected but unconfirmed UTIs and in 92.3% of confirmed UTIs.

**Conclusions:**

Using a stringent definition, only 11.7% of our suspicions were confirmed. Besides acute dysuria and suprapubic pain, we were not able to prove that any other clinical sign/symptom or POCT CPR adds useful information to the UTI diagnosis. We confirmed the findings of earlier research that urine dipstick tests are useful in ruling out UTIs and identified a potential overuse of antimicrobials in our NH population.

**Supplementary Information:**

The online version contains supplementary material available at 10.1186/s12877-022-02866-2.

## Background

Urinary tract infections (UTIs) are one of the most common infections in older persons living in nursing homes (NHs). It is estimated that the error rate of a UTI diagnosis based solely on clinical criteria is 33% in a general population [1]. A much higher error rate is to be expected in older persons as they often present infections in an atypical way. Signs/symptoms can be more subtle (e.g. absence or reduced intensity of fever) or less specific (e.g. acute change in mental or functional status) in this age group compared to younger adults. Older adults also less frequently present typical symptoms of a UTI such as dysuria and frequency [2]. A meta-analysis by Gbinigie et al. tried to evaluate the diagnostic value of different signs/symptoms in older outpatients. The results of this meta-analysis are however difficult to interpret due to heterogeneity in the applied definitions of a UTI in the included studies [3]. Hence, this topic needs further study.

As the diagnosis cannot be solely based on signs/symptoms, additional diagnostic testing is required to improve antimicrobial prescribing in NH residents suspected of having a UTI. Bacteriological urine culture has become a standard test in the diagnosis of UTI and is also recommended for older persons as their microbiology differs from the young [4]. To minimize contamination the urine specimen should be appropriately collected. This is not straightforward in older persons who suffer from incontinence and/or are physically or cognitively impaired [5–7]. In addition, many older adults have bacteria in their urine (bacteriuria). The prevalence varies from 25 to 50% in institutionalized women and from 15 to 40% in men [7, 8]. A Belgian study confirmed this finding and even reported a 80–90% prevalence of asymptomatic bacteriuria in a subpopulation of female NH residents with urinary incontinence or with a high degree of dependence and disorientation [9]. Despite trials reporting no benefits and even potential harms (increased risk of adverse events, costs and antimicrobial resistance) with antimicrobial treatment of asymptomatic bacteriuria, this practice continues to be prevalent in NHs [10–12]. Up to 50% of all antimicrobial treatments in NHs are still prescribed to asymptomatic residents [12]. The interpretation of bacteriuria as symptomatic in the presence of nonspecific symptoms, the uncertainty of physicians about the significance of a positive urine culture result and concerns over liability of nurses and physicians play herewith an important role [13].

A second commonly used instrument for diagnostic testing is the dipstick. A meta-analysis concluded that urine dipstick tests are only useful to exclude the presence of an infection provided the results for both nitrites and leukocyte-esterase are negative [14]. A cross-sectional study confirmed this in a NH setting [15].

In an attempt to increase the precision of diagnostic algorithms several biomarkers are being tested. Increased levels of some inflammatory markers (e.g. C-reactive protein (CRP), interleukins) are seen in UTIs but their diagnostic or prognostic utility has not been documented so far. Only few studies have explored these biomarkers in an elderly population like NH residents [16, 17].

Inflammatory markers could play an important role in diagnosing serious infections in older persons in whom clinical signs are often presented atypically. CRP, an acute phase protein produced by the liver in response to any infection or inflammation, has been studied extensively but its role in predicting severe infections in older adults remains unclear [2, 18, 19]. Point-of-care testing (POCT) allows CRP to be tested at the bedside. The added value of this method could be substantial in NHs. These facilities, mainly providing accommodation to frail older adults, often have a more restricted access to clinical laboratories and it sometimes takes a long time to obtain the final test result. The major advantage of POCT is that it provides results in only a few minutes, allowing physicians to make clinical decisions more quickly and efficiently [20]. Older patients could benefit from such a timely bedside diagnosis as they tend to deteriorate more rapidly from infections [2]. In addition, POCT CRP requires only a droplet of blood and may therefore be easier to use in older persons from whom it is not always easy to obtain traditional blood samples. Nonetheless, a restriction of CRP is that it can increase also as a result of other medical conditions or infectious diseases.

At present, there is no standard diagnostic procedure for UTIs in Belgian NH residents. Each visiting general practitioner can decide on the proper management for his or her patients. A survey revealed that less than half of Belgian NHs routinely order urine cultures and that only 30% routinely use urine dipstick tests [21]. To our knowledge, no POCT CRP tests are used to diagnose UTIs or other infections in our NH residents.

In the current study, we imposed all three diagnostic tests whenever there was a suspicion of a UTI. We aimed to investigate the diagnostic value of signs/symptoms and urine dipstick tests in identifying UTIs in NH residents and explored whether POCT CRP can play an additional role in the diagnosis of UTIs in older adults.

## Methods

### Study design and setting

This was a prospective diagnostic accuracy study conducted in Belgian NHs in 2016. Facilities where at least one of the participating microbiological laboratories could collect urine samples (*n* = 59) were invited to voluntarily participate. No financial incentives were given.

### Participants

NHs were asked to complete a resident questionnaire and perform diagnostic tests in each eligible residents with a suspected UTI during a predefined period of three months (observation period). A resident was eligible if he/she was older than 65 years and agreed to voluntarily take part in the study. Residents had to be excluded if they were not living fulltime in the facility (e.g. day centre visitors) or if he/she was in the NH for short-stay only.

### Questionnaires and diagnostic tests

The resident questionnaire collected (1) general information (e.g. date of UTI suspicion, person who suspected the UTI), (2) resident characteristics (e.g. gender, birth year, length of stay in the NH), (3) resident’s care load and risk factors (e.g. incontinence, urinary catheter use), (4) signs/symptoms of a UTI and general infection signs and (5) diagnostic test results (described hereafter). We used the Belgian adaption of the index of independence in activities of daily living (ADL), i.e. KATZ score, to express the residents’s care load. It classifies residents based on their level of dependency based on ADL-performance and orientation in time and space [22, 23].

NH staff were asked to thick all signs/symptoms suggesting a UTI in the questionnaire before performing the diagnostic tests. Firstly, a urine sample had to be taken before initiating antimicrobial therapy. If the condition of the resident allowed it, a clean midstream sample was preferred. Alternatively, spontaneously voided urine, urine collected through in-and-out catheterization or a catheter specimen were allowed. Immediately after collecting this sample, a test strip (Combur2 Test® LN; Roche Diagnostics, Switzerland) had to be submerged in the urine. According to the instructions of the manufacturer the result of the nitrite and leukocyte esterase test had to be read and documented in the resident questionnaire. Afterwards, the urine sample had to be correctly identified (study number of the resident, date and time of collection and method of sampling) and sent to one of the two microbiological laboratories participating in this study. If the urine sample could not be transported immediately to the laboratory, the urine had to be refrigerated at four degrees Celsius. Urinalysis, urine culture and susceptibility testing were performed in the microbiological laboratory. Immediately after or before urine collection, POCT CRP was performed within the NH. Finger sticks were done using safety lancets (Sarstedt, Germany). Blood drops were pipetted into the capillary of the CRP test cartridges and analysed using the Afinion AS100 Analyzer (Abbott, formally Alere Health). The test required a 1.5 µL blood volume and had a measuring range for CRP of 5–200 mg/L. Results below 5 mg/L were displayed as < 5 mg/L, results above 200 as > 200 mg/L. Assay time was about 4 min. For internal quality control, a positive sample had to be measured regularly by the NH staff to confirm the efficacy and correct performance of the test.

Each NH received all necessary study documents and materials (e.g. dipstick tests, CRP test cartridges and finger sticks). One Afinion AS100 Analyzer was made available per NH ward.

### Training

Before the start of the study, a training session was organized in each facility by a member of the research team. During these sessions, the methodology was elaborated and practical and ethical aspects were highlighted. NH staff were shown how to perform POCT CRP and quality controls by means of a hands-on demonstration by the research team who in their turn were trained by a representative of Alere Health.

### Applied definitions

In the present study, a UTI suspicion was defined as each sign or symptom that caused a physician, (head) nurse, nursing assistant or other person (e.g. the resident itself) to suspect a UTI. UTIs were confirmed if they matched the surveillance definition as described in Stone et al., i.e. presence of a minimum number of signs/symptoms and a positive urine culture, taking into account whether or not the resident had an indwelling catheter (Table 1) [24]. Contaminated samples were considered negative. Results of the antimicrobial susceptibility testing, including multidrug resistance (MDR; i.e. intermediate susceptibility and resistance) to ≥ 1 agent in ≥ 3 antimicrobial categories) were explored [25]. Recurrent UTI suspicions within 14 days were considered as one infection episode. Antimicrobials were classified according to the Anatomical Therapeutic Chemical (ATC) Classification System of the World Health Organization [26].

**Table 1:** Surveillance definition for urinary tract infections according to Stone MD et al. [24]

For residents without an indwelling catheter, both the signs/symptoms (A) and urine culture (B) criteria must be met **A. Signs/symptoms** At least one of the following (1, 2 or 3) sign or symptom subcriteria: 1. Acute dysuria or acute pain, swelling, or tenderness of the testes, epididymis, or prostate 2. Fever^a^ OR leukocytosis^b^ and at least one of the following localizing urinary tract subcriteria: Acute costovertebral angle pain or tenderness Suprapubic pain Gross haematuria New or marked increase in frequency New or marked increase in urgency New or marked increase in incontinence 3. In the absence of fever or leukocytosis, then two or more of the following localizing urinary tract subcriteria: Suprapubic pain Gross haematuria New or marked increase in frequency New or marked increase in urgency New or marked increase in incontinence **B. Urine culture** One of the following (1 or 2) microbiological subcriteria: 1. At least 10^5^ colony forming units per millilitre (cfu/mL) of no more than 2 species of microorganisms in a voided urine sample 2. At least 10^2^ cfu/mL of any number of organisms in a specimen collected by in-and-out catheter	For residents with an indwelling catheter, both the signs/symptoms (A) and urine culture (B) criteria must be met **A. Signs/symptoms** At least one of the following (1, 2, 3 or 4) sign or symptom subcriteria: 1. Fever, rigors or new-onset hypotension with no alternate site of infection 2. Either acute change in mental status or acute functional decline, with no alternate diagnosis and leukocytosis 3. New-onset suprapubic pain or costovertebral angle pain or tenderness 4. Purulent discharge around the catheter or acute pain, swelling, or tenderness of the testes, epididymis, or prostate **B. Urine culture** Urinary catheter specimen culture with at least 10^5^ cfu/mL of any organism(s) Note: Urinary catheter specimens for culture should be collected following replacement of the catheter (if current catheter has been in place for > 14 days)

*Cfu/mL* Colony-forming units per millilitre; ^a^Fever: (1) single oral temperature of > 37.8° C (Celsius) or (2) repeated oral temperatures > 37.2° C or rectal temperatures > 37.5° C or (3) single temperature > 1.1° C over baseline from any site (oral, tympanic, axillary); ^b^Leukocytosis: (1) neutrophilia (> 14 000 leukocytes/mm^3^) or (2) left shift (> 6% bands or ≥ 1 500 bands/mm^3^)

### Sample size calculation

To calculate our sample size, we used the method of Flahault et al. for diagnostic studies and applied a prevalence of 1.30% which was found in a European point prevalence survey of healthcare-associated infections and antimicrobial use in long-term care facilities (LTCFs) [27, 28]. Given the binary (yes/no) form of the outcome measure, a binomial distribution was used. In order to obtain a sensitivity of 95% and a minimum acceptable lower 95% confidence interval (CI) of 75%, 34 residents with a UWI (cases) were needed. Consequently, according to the formula N_controls_ = N_cases_ [(1-prevalence) / prevalence] 2581 residents without UTIs (controls) were needed [27]. We therefore aimed to include 2600 residents.

### Statistical analyses

Stata version SE/16.1 (StataCorp, USA) was used for descriptive analyses and logistic regression. In order to calculate the incidence of UTIs per 10 000 resident-days in the participating NHs, the number of confirmed UTIs was multiplied by 10 000 and divided by the number of resident-days during the study period, which was estimated by multiplying the number of eligible residents present at the start of the study with 90 days (3 months). As the number of deaths during the registration period was limited and Belgian NH beds are quickly filled following a resident’s death, we did not correct for this.

Predictors for both confirmed UTIs and positive urine cultures only were calculated using univariate logistic regression and expressed in odds ratios (OR) and their 95% CI. A *p*-value of 0.05 was chosen as the cut-off point for statistical significance. Diagnostic test performances, i.e. sensitivity, specificity, positive likelihood ratios (LR +) and negative likelihood ratios (LR-) and their 95% CIs were calculated for individual signs/symptoms and test results using an online evidence-based medicine diagnostic test calculator [29]. When these calculations were not possible due to an empty cell, a continuity correction of 0.5 was added into each cell of the 2 × 2 table. Dumbbell plots (made in Microsoft Excel, 2016) were used to present the pre-test probability (UTI prevalence) and the post-test probability (derived from the LRs) of UTI given the presence (positive post-test probability) or absence (negative post-test probability) of the sign or symptom or a positive test. Signs/symptoms were found helpful in ruling in or out a UTI when the LRs 95% CI did not include de value of 1.0, respectively.

## Results

### Participation

Eleven NHs participated in the study, totalling 1 263 eligible residents. The characteristics of this population can be consulted in Additional file 1.

#### UTI suspicions and triggering signs/symptoms

In 115 residents (9.11%; 80.0% female) at least one UTI was suspected during the three-month observation period. Twenty residents had two UTI suspicions and one resident had three suspicions. The median time between two UTI suspicions was 33.5 days (interquartile range (IQR): 23.0–39.0). In total, 137 UTI suspicions were reported.

**Fig. 1:**
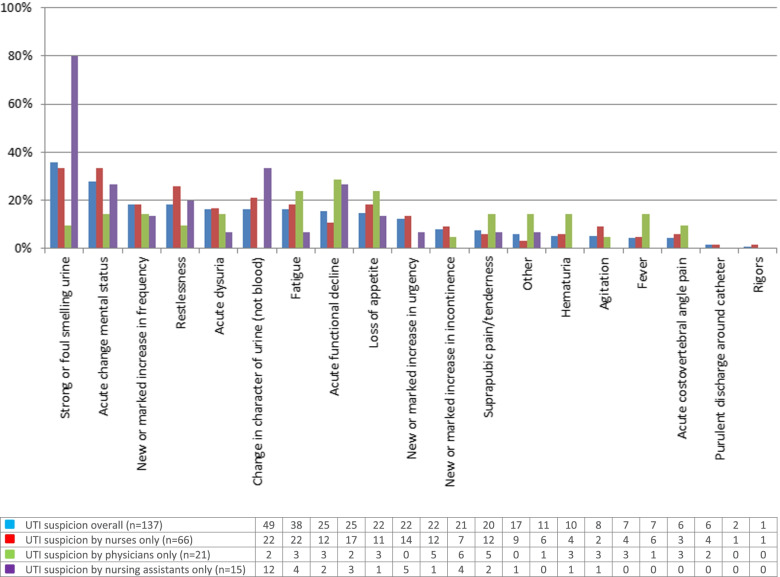
Signs/symptoms triggering suspicion of a UTI in Belgian nursing home residents. *UTI* Urinary tract infection

Figure 1 presents the signs/symptoms most frequently reported overall and by type of caregiver. On average, 2.40 signs/symptoms (maximum: 8) per UTI suspicion were reported. For four suspicions, no signs/symptoms were reported (missing information). Strong or foul smelling urine (35.8%), acute change in mental status (27.7%), new or marked increase in frequency (18.2%) and restlessness (18.2%) mainly triggered a UTI suspicion. Acute dysuria was reported in 16.1% of the cases. The group of “other” reported signs/symptoms included among others overall body pain, vomiting, hyperglycaemia and sticky discharge. Acute pain, swelling or tenderness of the testes, epididymis or prostate, leukocytosis and new-onset hypotension were not reported. In less than half of all UTI suspicions (46.7%), typical UTI symptoms (i.e. acute dysuria, new or marked increase in frequency, urgency or incontinence, suprapubic pain/tenderness and/or acute costovertebral angle pain) was reported.

### Urine culture results

Although the study protocol required a urine culture to be performed after each UTI suspicion, there were 13 results missing (9.49%). When a urine culture was performed (*n* = 124) the result was positive, negative or contaminated in 50.8% (*n* = 63), 21.8% (*n* = 27) and 27.4% (*n* = 34) of the cases, respectively.

The most frequently isolated microorganisms and their non-susceptibility profile can be found in Additional file 2. Of all isolates for which the status was determined, 28.1% were MDR.

### UTI confirmation

When applying the UTI surveillance definition (Table 1) to the 137 suspicions, the definition for residents with an indwelling catheter had to be used 13 times (9.49%). It concerned either a transurethral (*n* = 10) or a suprapubic catheter (*n* = 3). In seven cases (53.8%), the required number of signs/symptoms were present to meet the first part of the definition. One patient had purulent discharge around the catheter and one had fever. Five residents experienced an acute change in mental status or acute functional decline. Of the latter, one resident also had new-onset suprapubic pain or tenderness. Only two residents with an acute change in mental status or acute functional decline also had a positive urine sample and therefore had a confirmed UTI (*n* = 2/13; 15.4%). The other urine samples were reported as contaminated.

In 28.2% of the 124 UTI suspicions in residents without an indwelling catheter, enough signs/symptoms were present to comply with the first criteria of the surveillance definition. In these 35 cases, the urine culture result was negative, contaminated or missing in 22.9% (*n* = 8), 20.0% (*n* = 7) and 17.1% (*n* = 6) of the cases, respectively. In fourteen cases (11.3%), both criteria of the UTI definition (i.e. enough signs/symptoms and a positive urine culture) were fulfilled. In these confirmed UTIs, acute dysuria was most frequently reported (78.6%, *n* = 11) followed by suprapubic pain (35.7%, *n* = 5) and new or marked increase in urgency (28.6%, *n* = 4) or frequency (21.4%, *n* = 3).

In total, 16 UTI episodes were confirmed in 14 different residents. The overall crude incidence was 1.41 confirmed UTIs per 10 000 resident-days (median: 1.30, IQR: 0–1.86) [see Additional file 3].

### Accuracy of individual signs/symptoms and diagnostic tests

**Fig. 2:**
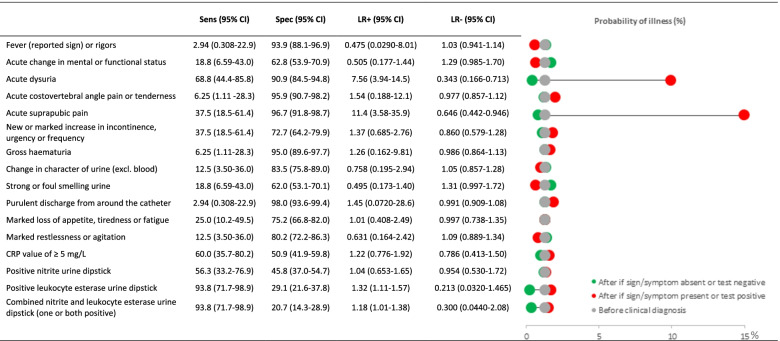
Performance of signs/symptoms, urine dipstick test and C-reactive protein in diagnosing confirmed UTIs^a^. ^a^*Confirmed UTIs*: Urinary tract infections corresponding to the definition of a UTI as described in an article of Stone MD et al., i.e. presence of enough urinary signs and/or symptoms and a positive urine culture [24], *CRP* C-reactive protein, *Sens* Sensitivity, *Spec* Specificity, *LR* + Positive likelihood ratio, *LR-* Negative likelihood ratio, *CI* Confidence interval, *Sens,* *Spec*, and their 95% CIs are expressed as percentages

Factors associated with confirmed UTIs and positive urine cultures are explored in Additional file 4. The individual test performances of the signs/symptoms and diagnostic tests in diagnosing a UTI can be found in Fig. 2. Only acute dysuria and acute suprapubic pain were significant predictors of a confirmed UTI. The sensitivity and specificity of the nitrite urine dipstick test alone was 56.3% and 45.8%, respectively. The sensitivity of the leucocyte esterase test alone equalled that of the combined nitrite and leucocyte esterase test (both 93.8%), but specificity was less than 30.0% for both (Fig. 2). The combined dipstick test had a 96.0% negative predictive value (95% CI: 80.5–99.3%). In just less than half of all UTI suspicions CRP was < 5 mg/L (*n* = 65/131; 49.6%). The CRP value was between 5 and 49 mg/L in 38.9% of the cases (*n* = 51), between 50–99 mg/L in 3.05% (*n* = 4) and > 100 mg/L in 8.0% (*n* = 11). The sensitivity and specificity of a positive CRP test (≥ 5 mg/L) was 60.0% and 50.9%, respectively.

### Antimicrobial treatment

Of all UTI suspicions, 64.0% (*n* = 71/111; 26 missing) were treated with antibiotics. The urine culture result was positive, negative, contaminated or missing in 47 (66.2%), 8 (11.3%), 9 (12.7%) and 7 (9.86%) of these treatments, respectively. In total, 92.3% of all confirmed UTIs were treated (*n* = 12/13; 3 missing).

Initial treatments were mainly reported as microbiologically-documented (*n* = 29/71; 40.8%). Empirical treatments (i.e. initiation of treatment before the causative pathogen is known) and prophylaxis accounted for 32.4% and 4.23% of the prescriptions, respectively. The indication for prescribing was unknown for 22.5% of the antibiotics.

The three most frequently used antibiotic classes were J01X ‘other antibacterials’ (50.7%), J01M quinolones (25.4%) and J01C beta-lactams/penicillins (12.7%).

## Discussion

This study looked at signs/symptoms triggering a UTI diagnosis and explored whether or not CRP could be of added value in the diagnosis of a UTI. Only 11.7% of our UTI suspicions corresponded to the applied definition of a confirmed UTI [24]. Strong or foul smelling urine and acute change in mental status mainly triggered a UTI suspicion. However, only acute dysuria and acute suprapubic pain were found significant and increased the post-test probability. The combined nitrite and leucocyte esterase urine had a high negative predictive value. Antimicrobials were prescribed in more than half of the events with a suspected but unconfirmed UTI, indicating a potential significant overuse in this NH population.

Conducting research in LTCFs can be challenging [30]. We also experienced some major limitations in our study. As there was no funding for this study (diagnostic material payed with own resources) we relied on the voluntary participation of NHs and on their staff to carry out the study. The observation period was limited to three months to make the study as feasible as possible and to limit the time investment. Three non-hospital laboratories were willing to take part in this study and invited all their surrounding NHs. Unfortunately, the majority of facilities were not eager to participate, either because they were not interested in the topic, had other projects going on or because they perceived the study as a significant time investment. In one region, we found no participants and therefore continued the study with only two microbiological laboratories. Because of this and a slower than expected inclusion of residents with a UTI suspicion, we reached only half of our intended sample size and only half of our expected confirmed UTI diagnoses. This led to larger confidence intervals around our estimates.

We applied a very stringent definition of confirmed UTIs, requiring a set of urinary signs/symptoms and a positive urine culture [24]. Some studies only demand a positive urine sample for their UTI diagnosis, an outcome which might not be appropriate given the high prevalence of asymptomatic bacteriuria in NHs [7–9]. Half of all urine cultures in our study were positive, while a quarter were contaminated and thus considered negative. Our protocol gave preference to a clean midstream sample. Notwithstanding training was provided, contaminated samples were to be expected. We included all residents in our study, even those with physical or cognitive impairments in whom it can be challenging to collect a clean-catch sample [6, 7].

There is lack of empirical data on which signs/symptoms are associated with UTIs in a geriatric population. Clinical features clearly indicating UTIs in adults are not always presented by older persons or they may not be able to communicate them. The association between non-specific signs, such as confusion or falls, and UTIs also remains unclear [31, 32]. Decision algorithms for UTIs in an older population, whether or not living in LTCFs, are hence often based on expert opinion and consensus [33]. The Stone criteria we used are a good example of this. These criteria are intended for surveillance and benchmarking purposes and are therefore designed to be highly specific, not sensitive [24, 33]. In the absence of formal evidence with respect to an optimal gold standard for UTI diagnosis in an older population, we felt that the Stone criteria were the best available and acceptable to use in this study. After all, a positive urine culture could not be used as a gold standard for a UTI because of the high prevalence of asymptomatic bacteriuria in this NH population [7–9].

A study of Walker et al. showed that there was a wide range of indicators, more subtle than the classic symptoms of a UTI (e.g. dysuria, frequency, urgency, suprapubic pain), that influenced the ordering of cultures and the prescribing of antibiotics in both general practitioners and nurses [13]. In our questionnaire, we therefore also included signs/symptoms which are not widely accepted and sometimes even controversial for the diagnosis of a UTI in an older adult, e.g. cloudy or foul smelling urine. Nonetheless, these changes in the characteristics of the urine are often reported by NH staff as triggers for UTI suspicions and we also wanted to explore if this was the case in our participating facilities [34, 35]. In our study, it were mostly nurses and nursing assistants who reported these signs.

We combined the nitrite and leukocyte esterase dipstick test results because studies showed that the urine dipstick test seems most useful in all populations, including older adults, to exclude the presence of infection if the results of both nitrites and leukocyte esterase are negative [14, 15].

In the absence of formal cut-off values for CRP, we used the most common cut-offs, i.e. < 5 mg/L (normal range) and ≥ 5 mg/L (elevated values). In real life practice, general practitioners are most likely to apply these cut-offs that are also stated in the manufacturer’s instructions. NH staff could report the CRP test result to the resident’s general practitioner, but we do not know whether this was always done and if this influenced UTI management.

One study found that a serum CRP cut-off value of 60 mg/L was the best predictor for early detection of bacterial infections, including UTIs, in an older hospitalised population. The mean CRP value in their non-infection control group was however 21.3 mg/L, suggesting that ageing and frailty can increase serum inflammation protein levels [36, 37]. In our study, we did not explore CRP levels in residents without UTI suspicions.

We identified one similar study that explored the sensitivity of POCT CRP and procalcitonin to diagnose UTIs in Dutch NH residents. They used the same POCT device, but applied a more stringent definition of a confirmed (‘true’) UTI, namely a positive urine leucocyte esterase test and symptom resolution in the course of adequate antibiotic treatment (i.e. proven susceptibility of isolated uropathogens to the administered antibiotic) in addition to the presence of at least two urinary or non-specific symptoms [38]. They registered 266 episodes of suspected UTIs (out of the targeted 440 episodes), and 19.8% of these met their definition of a ‘true’ UTI, a percentage slightly higher than ours (11.7%). With an empirically estimated cut-off of 6.5 mg/L, their sensitivity (57.2%, 95% CI: 48.9–65.4%) and specificity (54.4%, 95% CI: 51.8–57.0%) of POCT CRP were similar to ours (60.0% and 50.9%, respectively) [38]. Signs/symptoms triggering UTI suspicion were not reported [39].

In this study, acute dysuria and acute suprapubic pain were the only symptoms found to be significant predictors of a UTI. Still, as UTI cases with such clear signs/symptoms are rare in NHs, the need for a test that helps identify UTIs in cognitively impaired residents or in older adults presenting the infection in an atypical way, remains. We hoped POCT CRP could help in these situations, but the sensitivity and specificity of this test were poor. Presumably, most infections in our study were lower UTIs causing only a limited inflammatory response and therefore low CRP levels.

The generalisability of our findings is limited by the low number of UTI cases in our study [40]. Analyses were not carried out on resident-level, but also included multiple confirmed UTIs or suspicions in the same resident. Moreover, our results are somewhat biased as explored symptoms were also part of the measured outcome (i.e. included in the definition of a confirmed UTI). Nonetheless, in the absence of a clear diagnostic test, a combination of signs/symptoms with a positive urine culture is the best gold standard to use for identifying UTIs.

Because of their practicality and the quick results, POCT could be of great value in a long-term care setting. Kuil et al. however concluded from their qualitative study that the inflammatory marker POCT could lead to new uncertainties. On the one hand, a negative test result could rule out UTI and justify withholding antibiotic treatment but doubts about the cause of the persisting symptoms can remain. On the other hand, a positive test could help rule in a UTI and justify antimicrobial treatment although, if signs/symptoms are not specific, another infection could be overlooked and inappropriately treated [41].

Additional studies exploring altered CRP cut-off values and novel urinary markers in older NH residents are highly needed. In their review, Horváth et al. identified promising biomarkers in UTIs that were already thoroughly studied (such as neutrophil gelatinase-associated lipocalin and cytokines) or that need further studies (e.g. urinary heparin binding protein). These biomarkers, apart from interleukins, were mostly assessed in a paediatric population [42].

## Conclusions

Using a stringent definition, only 11.7% of our suspicions were confirmed as UTIs. Besides acute dysuria and suprapubic pain, we were not able to show that any other clinical sign/symptom adds useful information to the UTI diagnosis. We confirm the finding of earlier research that urine dipstick tests are useful in ruling out UTIs in a NH population. In our study, POCT CRP hardly contributed to the diagnosis of a UTI in older NH residents using a cut-off value of 5 mg/L. It could be useful to have prospective trials exploring other CRP cut-off values or novel urinary markers in older NH residents.

## Supplementary Information

**Additional file 1.** Characteristics of the participating nursing homes and their eligible residents.

**Additional file 2.** Microorganisms isolated from nursing home residents with a positive urine culture and their non-susceptibility.

**Additional file 3.** Urinary tract infection (UTI) suspicions, positive urine cultures, confirmed UTIsa and incidence estimates by nursing home.

**Additional file 4.** Factors associated with confirmed urinary tract infectionsa using univariate logistic regression.

## Data Availability

The datasets used and/or analysed during the current study are available from the corresponding author on reasonable request.

## Abbreviations

ADL: Activities of daily living
ASB: Asymptomatic bacteriuria
CART: Classification and regression tree analyse
CI: Confidence interval
CRP: C-reactive protein
IQR: Interquartile range
LR-: Negative likelihood ratio
LR +: Positive likelihood ratio
LTCF: Long-term care facility
MDR: Multidrug resistance
NH: Nursing home
OR: Odds ratio
POCT: Point-of-care testing
UTI: Urinary tract infection

## References

[1] Schmiemann G, Kniehl E, Gebhardt K, Matejczyk MM, Hummers-Pradier E (2010). The diagnosis of urinary tract infection: A systematic review. Dtsch Arztebl Int.

[2] Van Duin D (2012). Diagnostic challenges and opportunities in older adults with infectious diseases. Clin Infect Dis.

[3] Gbinigie OA, Ordóñez-Mena JM, Fanshawe TR, Plüddemann A, Heneghan C (2018). Diagnostic value of symptoms and signs for identifying urinary tract infection in older adult outpatients: systematic review and meta-analysis. J Infect.

[4] Norman DC, Yoshikawa TT, Norman DC (2009). Clinical features of infection. Infectious disease in the aging a clinical handbook.

[5] Latour K, Plüddemann A, Thompson M, Catry B, Price CP, Heneghan C (2013). Diagnostic technology: alternative sampling methods for collection of urine specimens in older adults. Fam Med Community Health.

[6] Brazier AM, Palmer MH (1995). Collecting clean-catch urine in the nursing home: obtaining the uncontaminated specimen. Geriatr Nurs.

[7] Nicolle LE, SHEA Long-Term-Care-Committee (2001). Urinary tract infections in long-term-care facilities. Infect Control Hosp Epidemiol.

[8] Nicolle LE (1997). Asymptomatic bacteriuria in the elderly. Infect Dis Clin North Am.

[9] Biggel M, Heytens S, Latour K, Bruyndonckx R, Goossens H, Moons P (2019). Asymptomatic bacteriuria in older adults: the most fragile women are prone to long-term colonization. BMC Geriatr.

[10] Nicolle L (2016). The Paradigm Shift to Non-Treatment of Asymptomatic Bacteriuria. Pathogens.

[11] Zalmanovici Trestioreanu A, Lador A, Sauerbrun-Cutler M-T, Leibovici L. Antibiotics for asymptomatic bacteriuria. Cochrane Database Syst Rev. 2015;4:CD009534. 10.1002/14651858.CD009534.pub2 PMC8407041 25851268

[12] Phillips CD, Adepoju O, Stone N, Moudouni DK, Nwaiwu O, Zhao H (2012). Asymptomatic bacteriuria, antibiotic use, and suspected urinary tract infections in four nursing homes. BMC Geriatr.

[13] Walker S, McGeer A, Simor AE, Armstrong-Evans M, Loeb M (2000). Why are antibiotics prescribed for asymptomatic bacteriuria in institutionalized elderly people? A qualitative study of physicians’ and nurses’ perceptions. CMAJ.

[14] Devillé WL, Yzermans JC, van Duijn NP, Bezemer PD, van der Windt DA, Bouter LM (2004). The urine dipstick test useful to rule out infections. A meta-analysis of the accuracy. BMC Urol.

[15] Sundvall PD, Gunnarsson RK (2009). Evaluation of dipstick analysis among elderly residents to detect bacteriuria: a cross-sectional study in 32 nursing homes. BMC Geriatr.

[16] Nanda N, Juthani-Mehta M (2009). Novel biomarkers for the diagnosis of urinary tract infections – a systematic review. Biomark Insights.

[17] Sundvall PD, Elm M, Ulleryd P, Mölstad S, Rodhe N, Jonsson L (2014). Interleukin-6 concentrations in the urine and dipstick analyses were related to bacteriuria but not symptoms in the elderly: a cross sectional study of 421 nursing home residents. BMC Geriatr.

[18] Singh T, Newman AB (2011). Inflammatory markers in population studies of aging. Ageing Res Rev.

[19] Bourdel-Marchasson I, Laksir H, Puget E (2010). Interpreting routine biochemistry in those aged over 65 years: a time for change. Maturitas.

[20] Drain PK, Hyle EP, Noubary F, Freedberg KA, Wilson D, Bishai WR (2014). Diagnostic point-of-care tests in resource-limited settings. Lancet Infect Dis.

[21] Latour K, De Lepeleire J, Jans B, Buntinx F, Catry B (2020). Diagnosis, prevention and control of urinary tract infections: a survey of routine practices in Belgian nursing homes. J Infect Prev.

[22] Katz S, Ford AB, Moskowitz RW, Jackson BA, Jaffe MW, Studies of illness in the aged (1963). The index of ADL: a standardized measure of biological and psychological function. JAMA.

[23] De Lepeleire J, Paquay L, Jacobs M (2005). De verschillende schalen voor activiteiten van het dagelijks leven (ADL) voor volwassenen in de Vlaamse gezondheidszorg. Huisarts Nu.

[24] Stone MD, Ashraf MS, Calder J, Crnich CJ, Crossley K, Drinka PJ (2012). Surveillance definitions of infections in long-term care facilities: Revisiting the McGeer criteria. Infect Control Hosp Epidemiol.

[25] Magiorakos AP, Srinivasan A, Carey RB, Carmeli Y, Falagas ME, Giske CG (2012). Multidrug-resistant, extensively drug-resistant and pandrug-resistant bacteria: an international expert proposal for interim standard definitions for acquired resistance. Clin Microbiol Infect.

[26] World Health Organization (WHO) Collaborating Centre for Drug Statistics Methodology. The ATC/DDD system: International language for drug utilization research. Oslo: WHO Collaborating Centre for Drug Statistics Methodology and the Norwegian Institute of Public Health; 2017.

[27] Flahault A, Cadilhac M, Thomas G (2005). Sample size calculation should be performed for design accuracy in diagnostic test studies. J Clin Epidemiol.

[28] European Centre for Disease Prevention and Control (ECDC) (2014). Point prevalence survey of healthcare-associated infections and antimicrobial use in European long-term care facilities April–May 2013.

[29] Knowledge Translation Program. Evidence-based medicine toolbox: diagnostic test calculator, 2017. https://ebm-tools.knowledgetranslation.net/calculator/diagnostic/. Accessed 17 Feb 2021.

[30] Lam HR, Chow S, Taylor K, Chow R, Lam H, Bonin K (2018). Challenges of conducting research in long-term care facilities: a systematic review. BMC Geriatr.

[31] Sundvall PD, Ulleryd P, Gunnarsson RK (2011). Urine culture doubtful in determining etiology of diffuse symptoms among elderly individuals: a cross-sectional study of 32 nursing homes. BMC Fam Prac.

[32] Mayne S, Bowden A, Sundvall PD, Gunnarsson R (2019). The scientific evidence for a potential link between confusion and urinary tract infection in the elderly is still confusing - a systematic literature review. BMC Geriatr.

[33] Nace DA, Drinka PJ, Crnich CJ (2014). Clinical uncertainties in the approach to long term care residents with possible urinary tract infection. J Am Med Dir Assoc.

[34] Juthani-Mehta M, Tinetti M, Perrelli E, Towle V, Van Ness PH, Quagliarello V (2007). Diagnostic accuracy of criteria for urinary tract infection in a cohort of nursing home residents. J Am Geriatr Soc.

[35] Juthani-Mehta M, Tinetti M, Perrelli E, Towle V, Van Ness PH, Quagliarello V (2008). Interobserver variability in the assessment of clinical criteria for suspected urinary tract infection. Infect Control Hosp Epidemiol.

[36] Liu A, Bui T, Van Nguyen H, Ong B, Shen Q, Kamalasena D (2010). Serum C-reactive protein as a biomarker for early detection of bacterial infection in the older patient. Age Ageing.

[37] Soysal P, Stubbs B, Lucato P, Luchini C, Solmi M, Peluso R (2016). Inflammation and frailty in the elderly: a systematic review and meta-analysis. Ageing Res Rev.

[38] Kuil SD, Hidad S, Fischer J, Harting J, Hertogh CM, Prins JM (2019). Sensitivity of point-of-care testing c reactive protein and procalcitonin to diagnose urinary tract infections in Dutch nursing homes: PROGRESS study protocol. BMJ Open.

[39] Kuil SD, Hidad S, Fischer J, Harting J, Hertogh CM, Prins JM, et al. Sensitivity of C-reactive protein and procalcitonin measured by Point-of-Care tests to diagnose urinary tract infections in nursing home residents: a cross-sectional study. Stat Methods Med Res. 2019;28(8):2455-74. 10.1177/0962280218784726. 10.1093/cid/ciaa1709 PMC8664473 33175147

[40] van Smeden M, Moons KG, de Groot JA, Collins GS, Altman DG, Eijkemans MJ, et al. Sample size for binary logistic prediction models: beyond events per variable criteria. Stat Methods Med Res. 2019;28:2455–74. 10.1177/0962280218784726. 10.1177/0962280218784726 PMC6710621 29966490

[41] Kuil SD, Schneeberger C, van Leth F, de Jong MD, Harting J (2020). “A false sense of confidence” The perceived role of inflammatory point-of-care testing in managing urinary tract infections in Dutch nursing homes: a qualitative study. BMC Geriatr.

[42] Horváth J, Wullt B, Naber KG, Köves B (2020). Biomarkers in urinary tract infections – which ones are suitable for diagnostics and follow up?. GMS Infect Dis.

