# Supplementary material for: Nursing home residents with suspected urinary tract infections: a diagnostic accuracy study
Source: BMC Geriatr. 2022 Mar 7;22:187. doi: 10.1186/s12877-022-02866-2 (PMC8903673; doi:10.1186/s12877-022-02866-2)
Supplement: Supplementary file 1 — Additional file 1. Characteristics of the participating nursing homes and their eligible residents. [file 12877_2022_2866_MOESM1_ESM.pdf]

Additional file 1 Characteristics of the participating nursing homes and their eligible residents

						Resident's care load (%)				
**Nursing home (NH)**	**Ownership**	**Microbiological laboratory analysing the urine samples**	**N of eligible residents (65+ years)**	**Median age (IQR; in years)**	**% male residents**	Physically in-dependent and oriented in time and space (category 1)	Physically in-dependent and disoriented[a] OR Mild physical dependence without disorientation (category 2)	Severe physical de-pendence without dis-orientation[a] (category 3)	Severe physical dependence and dis-orientation[a] OR Diagnosis of dementia (category 4)	Unknown
A	Private not-for-profit	Lab A	119	87 (82-91)	26.1	2 (1.68)	56 (47.1)	20 (16.8)	41 (34.5)	0 (0)
B	Public	Lab A	40	-	-	-	-	-	-	40 (100)
C	Private not-for-profit	Lab B	88	86 (81-92)	39.8	1 (1.14)	36 (40.9)	9 (10.2)	28 (31.8)	14 (15.9)
D	Private not-for-profit	Lab A	65	85 (83-89)	20.0	10 (15.4)	32 (49.2)	7 (10.8)	15 (23.1)	1 (1.54)
E	Private not-for-profit	Lab B	77	88 (85-93)	27.3	10 (13.0)	30 (39.0)	16 (20.8)	21 (27.3)	0 (0)
F	Private not-for-profit	Lab B	179	88 (83-92)	31.3	14 (7.82)	46 (25.7)	31 (17.3)	88 (49.2)	0 (0)
G	Private not-for-profit	Lab B	167	85 (80-90)	26.4	2 (1.20)	3 (1.80)	1 (0.599)	1 (0.599)	160 (95.8)
H	Public	Lab A	70	86 (82-90)	28.6	2 (2.86)	32 (45.7)	13 (18.6)	23 (32.9)	0 (0)
I	Private not-for-profit	Lab B	94	85 (78-90)	22.3	17 (18.1)	43 (45.7)	7 (7.45)	25 (26.6)	2 (2.13)
J	Public	Lab B	205	85 (78-90)	28.3[b]	32 (15.6)	85 (41.5)	15 (7.32)	73 (35.6)	0 (0)
K	Public	Lab B	159	87 (83-91)	29.6	19 (12.0)	71 (44.7)	17 (10.7)	52 (32.7)	0 (0)
**Total**			**1 263**	**87 (82-91)**	**27.8**[b]	**109 (8.63)**	**434 (34.4)**	**136 (10.8)**	**367 (29.1)**	**217 (17.2)**

[a] disorientation: disorientation in time and space; [b] 21 missing values; IQR = interquartile range
