# Supplementary material for: Nursing home residents with suspected urinary tract infections: a diagnostic accuracy study
Source: BMC Geriatr. 2022 Mar 7;22:187. doi: 10.1186/s12877-022-02866-2 (PMC8903673; doi:10.1186/s12877-022-02866-2)
Supplement: Supplementary file 2 — Additional file 2. Microorganisms isolated from nursing home residents with a positive urine culture and their non-susceptibility. [file 12877_2022_2866_MOESM2_ESM.pdf]

**Additional file 2** Microorganisms isolated from nursing home residents with a positive urine culture and their non-susceptibility

| Microorganism | Number of isolates (%) | Tetracycline | Ampicillin | Piperacillin/tazobactam | Amoxicillin/Clavulanic acid | Oxacillin | Cefuroxime axetil | Cefotaxime | Ceftazidime | Cefepime | Cotrimoxazole | Gentamicin | Ciprofloxacin | Norfloxacin | Levofloxacin | Nitrofurantoin | Fosfomycin | Vancomycin | Number of MDR (%) |
|---|---|---|---|---|---|---|---|---|---|---|---|---|---|---|---|---|---|---|---|
| | | Number of non-susceptible isolates* | | | | | | | | | | | | | | | | | |
| **Urine cultures in female residents (n=54)** | | | | | | | | | | | | | | | | | | | |
| *Escherichia coli* | 32 (59.3) | | 16 | 4/14 | 9/17 | | 9 | 3/8 | | 3/8 | 11 | 5/29 | 14 | 2/3 | 2/3 | 0 | 2 | | 12 (37.5) |
| *Klebsiella pneumoniae* | 9 (16.7) | | | 1/8 | 0 | | 1 | 0/1 | | 0/1 | 0 | 0/8 | 0/8 | 0/1 | 0/1 | 5 | 2 | | 1 (11.1) |
| *Proteus mirabilis* | 5 (9.26) | | 0 | | 0/1 | | 0 | | | | 2 | 0/4 | 3 | 1/1 | 1/1 | | 2 | | 1 (20.0) |
| *Enterococcus faecalis* | 4 (7.41) | 4 | 0 | | | | | | | | | | | | 1 | 1 | | | 0 (0) |
| *Staphylococcus epidermidis* | 2 (3.70) | 0 | | | | 1 | | | | | 0 | | | | | | | 0 | |
| *Proteus vulgaris* | 1 (1.85) | | 1 | | 0 | | | | | | 0 | | 0 | 0 | | 1 | 0 | | 0 (0) |
| *Corynebacterium striatum* | 1 (1.85) | | | | | | | | | | | | | | | | | | |
| **Urine cultures in male residents (n=14)** | | | | | | | | | | | | | | | | | | | |
| *Escherichia coli* | 6 (42.9) | | 3 | 1/2 | 0/3 | | 4 | 0/3 | | 0/3 | 2 | 0/5 | 5 | 1/1 | 1/1 | 2 | 0 | | 3 (50.0) |
| *Proteus mirabilis* | 2 (14.3) | | 0 | | | | 0 | | | | 1 | 0 | 0 | | | | 1 | | 0 (0) |
| *Pseudomonas aeruginosa* | 2 (14.3) | | | 1 | | | | | 0 | | | 0 | 0 | | | | | | 0 (0) |
| *Enterococcus faecalis* | 2 (14.3) | 2 | 0 | | | | | | | | | | | | 1 | 0 | | | 0 (0) |
| *Klebsiella pneumoniae* | 1 (7.14) | | | 1 | 1 | | 1 | 1 | | 1 | 1 | 1 | 1 | | | 1 | 0 | | 1 (100) |
| *Aerococcus urinae* | 1 (7.14) | | | | | | | | | | | | | | | | | | |

*non-susceptible: intermediate susceptibility + resistance; MDR: multidrug resistance or non-susceptible to ≥1 agent in ≥3 antimicrobial categories
