# Supplementary material for: Nursing home residents with suspected urinary tract infections: a diagnostic accuracy study
Source: BMC Geriatr. 2022 Mar 7;22:187. doi: 10.1186/s12877-022-02866-2 (PMC8903673; doi:10.1186/s12877-022-02866-2)
Supplement: Supplementary file 3 — Additional file 3. Urinary tract infection (UTI) suspicions, positive urine cultures, confirmed UTIsa and incidence estimates by nursing home. [file 12877_2022_2866_MOESM3_ESM.pdf]

Additional file 3 Urinary tract infection (UTI) suspicions, positive urine cultures, confirmed UTIs[a] and incidence estimates by nursing home

Nursing home	N of eligible residents (65+ years)	Number of residents with at least one UTI suspicion (% of eligible residents)	Number of residents with at least one positive urine culture (% of eligible residents)	Number of residents with at least one confirmed UTI* (% of eligible residents)	Number of UTI suspicions	Number of positive urine cultures (% of UTI suspicions)	Number of confirmed UTIs[a] (% of UTI suspicions)	Incidence of confirmed UTIs per 10 000 resident-days
A	119	8 (6.72)	4 (3.36)	0 (0)	9	4 (44.4)	0 (0)	0
B	40	3 (7.50)	0 (0)	0 (0)	3	0 (0)	0 (0)	0
C	88	6 (6.82)	3 (3.41)	1 (1.14)	7	4 (57.1)	1 (14.3)	1.26
D	65	5 (7.69)	3 (4.62)	1 (1.54)	6	4 (66.7)	1 (16.7)	1.71
E	77	17 (20.1)	9 (11.7)	2 (2.60)	21	11 (52.4)	2 (9.52)	2.89
F	179	18 (10.1)	10 (5.59)	3 (1.68)	22	10 (45.5)	3 (13.6)	1.86
G	167	7 (4.19)	3 (1.80)	1 (0.599)	9	4 (44.4)	2 (22.2)	1.33
H	70	4 (5.71)	0 (0)	0 (0)	4	0 (0)	0 (0)	0
I	94	20 (21.3)	12 (12.8)	2 (2.13)	23	14 (60.9)	2 (8.70)	2.36
J	205	7 (3.41)	3 (1.46)	1 (0.488)	10	3 (30.0)	1 (10.0)	0.542
K	159	20 (12.6)	8 (5.03)	3 (1.89)	23	9 (39.1)	4 (17.4)	2.80
**Total**	**1 263**	**115 (9.11)**	**55 (4.35)**	**14 (1.11)**	**137**	**63 (46.0)**	**16 (11.7)**	**1.41**

[a]Confirmed UTIs: UTIs corresponding to the definition of a UTI as described in an article of Stone MD et al., i.e. presence of enough urinary signs and/or symptoms and a positive urine culture [24].
