# Supplementary material for: Nursing home residents with suspected urinary tract infections: a diagnostic accuracy study
Source: BMC Geriatr. 2022 Mar 7;22:187. doi: 10.1186/s12877-022-02866-2 (PMC8903673; doi:10.1186/s12877-022-02866-2)
Supplement: Supplementary file 4 — Additional file 4. Factors associated with confirmed urinary tract infectionsa using univariate logistic regression. [file 12877_2022_2866_MOESM4_ESM.pdf]

Additional file 4 Factors associated with confirmed urinary tract infections[a] using univariate logistic regression

	Confirmed urinary tract infections (UTIs)			
	No of residents			
	without a UTI	with a UTI	OR (95% CI)	p-value
**Age**				
65-74 years	4	2	Ref.	
75-84 years	37	8	0.4 (0.1-2.8)	p=0.38
85+ years	80	6	0.2 (0.0-1.0)	p=0.049
**Gender**				
Female	98	14	Ref.	
Male	23	2	0.6 (0.1-2.9)	p=0.53
**Resident's care load**[b]				
Category 1	6	3	Ref.	
Category 2	37	7	0.4 (0.1-1.9)	p=0.24
Category 3	19	3	0.3 (0.0-2.0)	p=0.22
Category 4	50	2	0.1 (0.0-0.6)	p=0.012
**Fever (reported sign) or rigors**				
No	114	16	Ref.	
Yes	7	0	/	/
**Acute change in mental or functional status**				
No	76	13	Ref.	
Yes	45	3	0.4 (0.1-1.4)	p=0.16
**Acute dysuria**				
No	110	5	Ref.	
Yes	11	11	22.0 (6.5-74.9)	p<0.001
**Acute costovertebral angle pain or tenderness**				
No	116	15	Ref.	
Yes	5	1	1.5 (0.2-14.1)	p=0.70
**Acute suprapubic pain**				
No	117	10	Ref.	
Yes	4	6	17.6 (4.2-72.6)	p<0.001
**New or marked increase in frequency, urgency or incontinence**				

No	88	10	Ref.	
Yes	33	6	1.6 (0.5-4.8)	p=0.40
**Gross hematuria**				
No	115	15	Ref.	
Yes	6	1	1.3 (0.1-11.4)	p=0.83
**Change in character of urine (excl. blood)**				
No	101	14	Ref.	
Yes	20	2	0.7 (0.2-3.4)	p=0.68
**Strong or foul smelling urine**				
No	75	13	Ref.	
Yes	46	3	0.4 (0.1-1.4)	p=0.14
**Purulent discharge around catheter**				
No	119	16	Ref.	
Yes	2	0	/	/
**Marked loss of appetite, tiredness or fatigue**				
No	91	12	Ref.	
Yes	30	4	1.0 (0.3-3.4)	p=0.99
**Marked restlessness or agitation**				
No	97	14	Ref.	
Yes	24	2	0.6 (0.1-2.7)	p=0.49
**Combined nitrite and leukocyte esterase dipstick test performed in the nursing home**				
Both negative	24	1	Ref.	
One or both positive	92	15	3.9 (0.5-31.1)	p=0.20
**C-reactive protein via point-of-care test**				
<5 mg/L	59	6	Ref.	
≥5 mg/L	57	9	1.6 (0.5-4.6)	p=0.43

[a] *Confirmed urinary tract infections (UTIs): UTIs corresponding to the definition of a UTI as described in an article of Stone MD et al, i.e. presence of enough urinary signs and/or symptoms and a positive urine culture [24];* [b] *Category 1: physically independent and oriented in time and space, category 2: physically independent and disoriented in time and space OR mild physical dependence without disorientation in time and space, category 3: severe physical dependence without disorientation in time and space, category 4: severe physical dependence and disorientation in time and space OR diagnosis of dementia; CI: confidence interval*
